# Human Papillomavirus Genital Infections among Men, China, 2007–2009

**DOI:** 10.3201/eid1906.111597

**Published:** 2013-06

**Authors:** Zhonghu He, Ying Liu, Yuan Sun, Long Fu Xi, Ke Chen, Yiqiang Zhao, Lei Gao, Fangfang Liu, Yaqi Pan, Tao Ning, Lixin Zhang, Hong Cai, Yang Ke

**Affiliations:** Key Laboratory of Carcinogenesis and Translational Research (Ministry of Education), Laboratory of Genetics, Peking University Cancer Hospital and Institute, Beijing, People’s Republic of China (Z. He, Y. Liu, Y. Sun, K. Chen, Y. Zhao, L. Gao, F. Liu, Y. Pan, T. Ning, H. Cai, Y. Ke);; University of Washington, Seattle, Washington, USA (L.F. Xi);; Anyang Cancer Hospital, Anyang, People’s Republic of China (L. Zhang)

**Keywords:** HPV, men, China, viruses, genital infection, human papillomavirus

## Abstract

To determine prevalence of genital human papillomavirus (HPV) infection among men in rural China, we analyzed genital swab specimens. Among 2,236 male residents of rural Henan Province, HPV infection prevalence was 17.5%. The most common oncogenic and nononcogenic types were HPV-16 and HPV-3, respectively. Infection was associated with younger age and multiple sex partners.

Human papillomavirus (HPV) is an etiologic agent of cervical cancer. Among men, genital HPV infection plays a role in the development of anogenital cancer (*1*). Estimates of HPV infection prevalence among heterosexual men, mostly from North America and Europe, vary substantially (3.5%–45%) (*1*,*2*). In the People’s Republic of China, estimated HPV prevalence among women varies by geographic location (*3*), but no such estimate is available for men. Therefore, we conducted this cross-sectional study to determine the prevalence of genital HPV infection in a large population of men in a rural province of China and to evaluate relevant factors.

## The Study

In 2007–2009, a population-based esophageal cancer cohort study was initiated in 9 villages in rural Anyang, Henan Province, China (*4*). This HPV investigation was added to the original cohort study in 6 of the 9 villages. Eligibility criteria were as follows: 1) male sex; 2) permanent residency in the target villages; 3) age 25–65 years; and 4) no history of cancer, cardiovascular disease, or mental disorder. Of 3,571 eligible men, 3,172 (89%) were enrolled. The main reason why the other 399, who were substantially younger, did not participate was loss of contact because they were employed outside Anyang.

Participants were interviewed, and exfoliated cells were collected from the penile shaft, glans penis, coronal sulcus, and scrotum by using saline-soaked swabs (*5*). The sampling procedure was identical for circumcised and uncircumcised men. To assess the adequacy of the specimens, we tested each specimen by PCR for the β-globin gene. Positive specimens were subsequently tested for 13 oncogenic and 37 nononcogenic types of HPV by using PCR-based direct sequencing with a pair of SPF1/GP6+ primers (*6*). Samples with ambiguous HPV typing signals were subjected to further cloning and sequencing.

To evaluate the associations between exposure variables and the presence of HPV DNA, we used logistic regression analysis with stepwise backward elimination at p>0.1. To examine the association across the ordered categorical variables, we used a trend test.

Of 3,172 specimens tested, 2,236 were positive for β-globin and were included in the analysis (median participant age 42 years; interquartile range 35–52 years). We excluded from the study men who were older and reported less risky sexual behavior than those who were included (Technical Appendix).

Of the 40 HPV types we tested for, we detected 36, including 13 oncogenic types. Overall prevalence of HPV infection was 17.5% (95% CI 16%–19%). Oncogenic HPV was detected in 140 (6.3%; 95% CI 5.3%–7.3%) specimens, and nononcogenic HPV was detected in 251 (11%; 95% CI 9.9%–13%) specimens. Among 15 HPV-positive specimens that had ambiguous direct sequencing signals and were further cloned and resequenced for genotyping, 3 had a second type and minor types were ignored. Among these infections (Table 1), the most common oncogenic type detected was HPV-16 (17.4%), followed by HPV-18 (7.2%). The most common nononcogenic type was HPV-3 (16.4%), followed by HPV-57 (7.9%).

**Table 1:** Type-specific proportions of HPV infection among 2,236 men from rural Henan Province, China, 2007–2009*

Genotype†	Genus and species	Positive, no. (%)‡
Oncogenic type§		140 (35.8)
HPV-16	*Alpha-9*	68 (17.4)
HPV-18	*Alpha-7*	28 (7.2)
HPV-58	*Alpha-9*	13 (3.3)
HPV-33	*Alpha-9*	8 (2.0)
HPV-45	*Alpha-7*	5 (1.3)
HPV-52	*Alpha-9*	4 (1.0)
HPV-66	*Alpha-6*	4 (1.0)
HPV-35	*Alpha-9*	3 (0.8)
HPV-68	*Alpha-7*	3 (0.8)
HPV-31	*Alpha-9*	1 (0.3)
HPV-51	*Alpha-5*	1 (0.3)
HPV-56	*Alpha-6*	1 (0.3)
HPV-59	*Alpha-7*	1 (0.3)
Nononcogenic type¶		251 (64.2)
HPV-3	*Alpha-2*	64 (16.4)
HPV-57	*Alpha-4*	31 (7.9)
HPV-87	*Alpha-3*	18 (4.6)
HPV-81	*Alpha-3*	17 (4.3)
HPV-11	*Alpha-10*	15 (3.8)
HPV-67	*Alpha-9*	12 (3.1)
HPV-90	*Alpha-14*	12 (3.1)
HPV-43	*Alpha-8*	11 (2.8)
HPV-75	*Beta-3*	10 (2.6)
HPV-54	*Alpha-13*	9 (2.3)
HPV-91	*Alpha-8*	9 (2.3)
HPV-94	*Alpha-2*	8 (2.0)
HPV-6	*Alpha-10*	6 (1.5)
HPV-30	*Alpha-6*	6 (1.5)
HPV-27	*Alpha-4*	5 (1.3)
HPV-40	*Alpha-8*	5 (1.3)
HPV-10	*Alpha-2*	4 (1.0)
HPV-62	*Alpha-3*	2 (0.5)
HPV-74	*Alpha-10*	2 (0.5)
HPV-84	*Alpha-3*	2 (0.5)
HPV-7	*Alpha-8*	1 (0.3)
HPV-29	*Alpha-2*	1 (0.3)
HPV-77	*Alpha-2*	1 (0.3)
Total		391 (100)

*HPV, human papillomavirus; *Alpha*, *Alphapapillomovirus*; *Beta*, *Betapapillomavirus*. †Of 391 HPV-positive specimens, 15 displayed ambiguous typing signals by direct sequencing of PCR product. Infection with >1 HPV type was detected in only 3 of 15 specimens that were tested by cloning and sequencing; the predominant type is shown. ‡Proportion. §Oncogenic types tested in this study included HPV-16, -18, -31, -33, -35, -39, -45, -51, -52, -56, -58, -59, and -66. ¶On the basis of latest published literature and our knowledge of the detection method of HPV DNA adopted in this study (SPF1/GP6+ mediated PCR and sequencing), nononcogenic types that could be tested for were HPV-3, -6, -7, -10, -11, -26, -27, -29, -30, -32, -37, -40, -42, -43, -44, -53, -54, -55, -57, -61, -62, -67, -68, -69, -70, -72, -74, -75, -77, -81, -82, -84, -85, -87, -90, -91, and -94.

Prevalence of infection of any or nononcogenic HPV types decreased significantly with participant’s increasing age (Table 2). Risk for infection with any HPV type was associated with being unmarried, having had multiple sex partners, and having had oral and anal sex. When the outcome was stratified by oncogenicity of HPV type, the association remained statistically significant for having had multiple sex partners.

**Table 2:** Selected demographic and behavior variables for genital HPV infection among 2,236 men from rural Henan Province, China, 2007–2009*

Patient variable	Any type HPV infection			Oncogenic HPV infection			Nononcogenic HPV infection	
	Crude OR (95% CI)†	Adjusted OR (95% CI)‡		Crude OR (95% CI)†	Adjusted OR (95% CI)‡		Crude OR (95% CI)†	Adjusted OR (95% CI)‡
	
Age, y								
25–35	1.0			1.0			1.0	
36–45	0.8 (0.6–1.0)			0.7 (0.4–1.1)			0.8 (0.6–1.1)	
46–55	0.8 (0.6–1.1)			0.9 (0.6–1.5)			0.8 (0.5–1.1)	
56–65	0.7 (0.5–1.0)			0.8 (0.5–1.3)			0.6 (0.4–1.0)	
p value for trend§	0.039			0.498			0.030	
Education level								
Illiterate, <1 y	1.0			1.0			1.0	
Primary school, 1–6 y	0.7 (0.4–1.4)			0.9 (0.3–2.4)			0.7 (0.3–1.4)	
Secondary school, 7–12 y	0.9 (0.5–1.6)			0.9 (0.3–2.2)			0.9 (0.4–1.9)	
College or above, >12 y	1.5 (0.5–4.3)			1.8 (0.4–8.3)			1.3 (0.4–4.8)	
p value for trend†	0.238			0.969			0.132	
Marital status								
Married or cohabiting	1.0	1.0		1.0	1.0		1.00	1.0
Never married, divorced, separated, or widowed	1.7 (1.1–2.6)	1.6 (1.0–2.5)		1.8 (1.0–3.5)	1.8 (0.9–3.4)		1.6 (0.9–2.7)	1.6 (0.9–2.8)
Type of employment								
Farming at home	1.0			1.0			1.0	
Working in local area	1.2 (0.9–1.5)			1.1 (0.7–1.6)			1.3 (0.9–1.8)	
Working outside local area	1.2 (0.9–1.6)			0.9 (0.6–1.5)			1.3 (0.9–1.9)	
Other	1.2 (0.6–2.4)			1.1 (0.4–3.1)			1.3 (0.6–2.9)	
p value for trend§	0.238			0.876			0.100	
Cigarette smoking								
Never	1.0			1.0			1.0	
Ever	1.0 (0.8–1.3)			0.9 (0.6–1.3)			1.1 (0.8–1.4)	
Alcohol consumption								
Never	1.0			1.0			1.0	
Ever	1.0 (0.8–1.3)			1.1 (0.8–1.6)			1.0 (0.7–1.3)	
Lifetime no. sex partners								
0–1	1.0	1.0		1.0	1.0		1.00	1.0
2	2.4 (1.6–3.6)	2.2 (1.5–3.4)		1.3 (0.6–2.7)	1.2 (0.6–2.6)		3.1 (2.0–4.8)	3.0 (2.0–4.7)
>3	2.0 (1.5–2.9)	1.8 (1.3–2.6)		1.9 (1.1–3.2)	1.9 (1.1–3.1)		2.1 (1.4–3.2)	1.9 (1.3–2.9)
p value for trend§	<0.001	<0.001		0.015	0.020		<0.001	<0.001
Oral or anal sex								
Never	1.0	1.0		1.0			1.0	
Ever	1.7 (1.2–2.4)	1.5 (1.0–2.1)		1.5 (0.9–2.6)			1.8 (1.2–2.6)	
Wash genitalia before sex								
Occasionally or never	1.0			1.0			1.0	
Frequently or every time	1.2 (0.9–1.6)			0.8 (0.5–1.4)			1.5 (1.1–2.1)	

*HPV, human papillomavirus; OR, odds ratio. †Crude ORs and 95% CIs derived by univariate logistic regression analysis. ‡Adjusted ORs and 95% CIs derived by multivariate logistic regression models including all the listed variables; backward method was used to select significant variables on the 0.10 level. §p values for trend derived by logistic regression analyses, treating categorical variables as continuous variables.

## Conclusions

This study of genital HPV in a large sample of adult men from rural China addresses the paucity of data on male genital HPV infection in Asia. Prevalence rates for any type of HPV and oncogenic HPV were lower among these men in China than among heterosexual men elsewhere (Asia–Pacific area and globally) (*2*,*7*). This discrepancy might be partly explained by the relatively more conservative sexual behavior and higher median age of participants in this study.

Among populations of similar age, prevalence of a specific HPV type is usually lower among men than among women (*1*). However, the 17.5% prevalence found in this study exceeds estimates for married women 15–59 years of age in China (14.8%–16.8%) (*8*). This finding is consistent with that of another study, which also reported higher HPV prevalence among men than among women (*9*). These inconsistent findings might be explained by differences in HPV type distribution between male genitalia and the female cervix and by variability of type-specific sensitivity in detection methods.

Although most studies of HPV infection in men worldwide have found no clear trend with regard to age (*1*), we found that infection mildly decreased with increasing age. A potential explanation for this age-related trend might be that in this population, young adults more commonly work for long periods outside the rural home area than do older adults (data not shown), and the increased mobility might be associated with more unprotected sexual behavior and consequent increased exposure to HPV (*10*).

Our finding that HPV-16 and -18 were the most commonly detected oncogenic HPV types is in keeping with findings of previous studies (*2*,*7*,*11*,*12*). However, our finding that HPV-3 and HPV-57 were the predominant nononcogenic types is in contrast to the findings of other studies that HPV-6 and HPV-11 were the most predominant (*2*,*11*,*13*). This discrepancy might partly be explained by the fact that in our in-house evaluation, the primer set SPF1/GP6+ was more sensitive for HPV-57 but less sensitive for HPV-11 than was GP5+/GP6+. Completely opposite to our findings, Dai et al. reported that among women in neighboring rural Shanxi Province, China, oncogenic types were more commonly detected than were nononcogenic types (*14*). One possible explanation is that in our study, a significant portion of the exfoliated cells were collected from skin tissue. Therefore, a number of cutaneous HPV types, which are nononcogenic for mucosal lesion (cervical cancer), could be detected. We believe this might have led to the higher proportion of nononcogenic HPV than oncogenic HPV detected in our study. Another possible explanation is that sequencing methods used in our study can detect more nononcogenic types, which escape identification in studies that use hybridization with preassigned probes.

In contrast to previously reported rates of infection (*7*,*12*), in our study, infection with multiple types was rare. The previous studies used hybridization, an efficient way to identify co-infections, for genotyping. However, we used sequencing instead of hybridization to maximize demonstration of the spectrum of HPV types. Because minor types would probably be covered by the predominant type in the sequencing process, sequencing would probably have resulted in underestimation of infection with multiple types.

The low proportion of β-globin positivity might have partly resulted from the lower efficiency of cell collection by use of a saline-moistened swab as opposed to other methods such as emery paper (*15*). Another possible explanation is that a certain proportion of cells collected from male genitalia are keratinized and contain less nucleated human DNA than cells collected from mucosal organs (e.g., the cervix) (*15*).

The age range of the male participants in this large study was broad. However, the biases potentially imposed by the nonparticipation of ≈400 younger men (because of mobility) and 936 older men (because of specimen inadequacy) must be noted. This nonparticipation of younger men might have neutralized the age-related association to some extent. Although circumcision is extremely rare in rural China, the lack of accurate data for this variable is another study limitation, which rendered subgroup analysis by circumcision status impossible.

As reported in other studies (*1*), having had multiple sex partners was associated with HPV infection in this population. This finding indicates that men with higher mobility (i.e., higher risk for multiple sex partners and unprotected sexual behavior) should receive more attention with regard to future HPV control in this region. 

Technical Appendix Characteristics of 3,172 men from rural Henan Province, by human papillomavirus β-globin status of genital specimens, 2007–2009.
