# Supplementary material for: Human Papillomavirus Genital Infections among Men, China, 2007–2009
Source: Emerg Infect Dis. 2013 Jun;19(6):992–5. doi: 10.3201/eid1906.111597 (PMC3713808; doi:10.3201/eid1906.111597)
Supplement: Technical Appendix — Characteristics of 3,172 men from rural Henan Province, by human papillomavirus β-globin status of genital specimens, 2007–2009. [file 11-1597-Techapp-s1.pdf]

Article DOI: http://dx.doi.org/10.3201/eid1906.111597

Human Papillomavirus Genital Infections among Men, China, 2007–2009

Technical Appendix

Technical Appendix Table. Characteristics of 3,172 men from rural Henan Province, by human papillomavirus β-globin status of genital specimens, 2007–2009*

Variable	β-Globin positive, no. (%), n = 2,236	β-Globin negative, no. (%), n = 936†	p value‡
Age, y			
25–35	636 (28.4)	166 (17.7)	0.003
36–45	778 (34.8)	307 (32.8)	
46–55	436 (19.5)	228 (24.4)	
56–65	386 (17.3)	235 (25.1)	
Median (IQR)	42 (35,52)	45 (38.56)	
Education†			
Illiterate (<1 y)	71 (3.4)	36 (4.16)	0.107
Primary school (1–6 y)	576 (27.6)	267 (30.8)	
High school (7–1 2y)	1.417 (67.8)	557 (60.3)	
College or above (>12 y)	26 (1.2)	7 (0.7)	
Marital status§			
Married or cohabiting	1.973 (94.4)	819 (94.6)	0.822
Never married, divorced, separated, or widowed	117 (5.6)	47 (5.4)	
Regular cigarette smoking§			
Never	743 (35.6)	331 (38.2)	0.169
Ever	1.347 (64.4)	535 (61.8)	
Regular alcohol consumption§			
Never	1415 (67.7)	602 (69.5)	0.336
Ever	675 (32.3)	264 (30.5)	
Lifetime no. female sex partners§			
0–1	1.753 (84.6)	756 (88.0)	0.047
2	127 (6.1)	44 (5.1)	
≥3	193 (9.3)	59 (6.9)	
Oral or anal sex§			
Never	1.890 (90.5)	807 (93.3)	0.013
Ever	199 (9.5)	58 (6.7)	

*IQR, interquartile range.

†β-Globin–negative specimens were considered inadequate, and the corresponding participants were excluded from analyses.

‡p values derived by χ^2 tests.

§Category entries in sum do not equal total sample size because of missing value.
